# Sociodemographic and occupational risk factors associated with the development of different burnout types: the cross-sectional University of Zaragoza study

**DOI:** 10.1186/1471-244X-11-49

**Published:** 2011-03-29

**Authors:** Jesús Montero-Marín, Javier García-Campayo, Marta Fajó-Pascual, José Miguel Carrasco, Santiago Gascón, Margarita Gili, Fermín Mayoral-Cleries

**Affiliations:** 1 Department of Psychiatry. University of Zaragoza, Zaragoza, Spain; 2 Faculty of Health and Sports. University of Zaragoza, Huesca, Spain; 3 Instituto Aragonés de Ciencias de la Salud (Aragon Health Sciences Institute), Zaragoza, Spain; 4 Department of Psychology and Sociology. University of Zaragoza, Teruel, Spain; 5 Institut Universitari d'Investigació en Ciències de la Salut (IUNICS). University of Balearic Islands, Spain; 6 Hospital Regional Universitario Carlos Hay, Malaga, Spain; 7 REDIAPP "Red de Investigación en Actividades Preventivas y Promoción de la Salud" RD06/0018/0017, Research Network on Preventative Activities and Health Promotion; 8 Psychiatry Service. Miguel Servet Hospital. Zaragoza, Spain

**Keywords:** burnout, subtypes, risk factors, BCSQ-36, university

## Abstract

**Background:**

Three different burnout types have been described: The "frenetic" type describes involved and ambitious subjects who sacrifice their health and personal lives for their jobs; the "underchallenged" type describes indifferent and bored workers who fail to find personal development in their jobs and the "worn-out" in type describes neglectful subjects who feel they have little control over results and whose efforts go unacknowledged. The study aimed to describe the possible associations between burnout types and general sociodemographic and occupational characteristics.

**Methods:**

A cross-sectional study was carried out on a multi-occupational sample of randomly selected university employees (n = 409). The presence of burnout types was assessed by means of the "Burnout Clinical Subtype Questionnaire (BCSQ-36)", and the degree of association between variables was assessed using an adjusted odds ratio (OR) obtained from multivariate logistic regression models.

**Results:**

Individuals working more than 40 hours per week presented with the greatest risk for "frenetic" burnout compared to those working fewer than 35 hours (adjusted OR = 5.69; 95% CI = 2.52-12.82; *p *< 0.001). Administration and service personnel presented the greatest risk of "underchallenged" burnout compared to teaching and research staff (adjusted OR = 2.85; 95% CI = 1.16-7.01; *p *= 0.023). Employees with more than sixteen years of service in the organisation presented the greatest risk of "worn-out" burnout compared to those with less than four years of service (adjusted OR = 4.56; 95% CI = 1.47-14.16; *p *= 0.009).

**Conclusions:**

This study is the first to our knowledge that suggests the existence of associations between the different burnout subtypes (classified according to the degree of dedication to work) and the different sociodemographic and occupational characteristics that are congruent with the definition of each of the subtypes. These results are consistent with the clinical profile definitions of burnout syndrome. In addition, they assist the recognition of distinct profiles and reinforce the idea of differential characterisation of the syndrome for more effective treatment.

## Background

Burnout syndrome has become an increasingly commonplace subject in the scientific literature. In the span of thirty-five years, since the appearance of the first clinical descriptions of the syndrome, we have been able to observe a considerable increase in the number of studies dealing with burnout. The growing interest of researchers in this psychosocial disorder is easy to understand. In a relatively short time, Western societies have experienced a series of economic, technological and social transformations that have impacted working conditions, often creating a greater vulnerability to stress.

Although different approaches have been considered regarding burnout syndrome, most authors accept that it is a uniform phenomenon, with specific aetiology and symptoms [1]. The most accepted definition is that described by Maslach, Schaufeli and Leiter [2]. According to their definition, burnout is the result of a prolonged exposure to chronic personal and interpersonal stressors on the job as determined by three dimensions: exhaustion, cynicism and professional inefficacy. "Exhaustion" is described as the feeling of not being able to offer any more of oneself at an emotional level; "cynicism" is refers to a distant attitude towards work, the people being served by it and among colleagues; and "inefficacy" describes the feeling of not performing tasks adequately and of being incompetent at work. However, burnout syndrome has been related historically to the presence of guilt feelings in the individual suffering from it [3-5]. According to Gil-Monte, this variable plays a major role in the development and chronification of the syndrome by means of a positive feedback mechanism in some of those affected [6,7].

Nevertheless, clinical experience suggests that the disorder manifests in several different ways, leading Farber to propose a preliminary classification system based on three different burnout types [1,8-13]. In this author's opinion, burnout is an experience during which individuals are aware of a considerable discrepancy between their contributions and rewards and between their invested efforts and the results obtained at work. This definition is the result of a phenomenological analysis of the syndrome, and it can be placed within the framework of the social exchange theory, according to which the establishment of reciprocal social relations is essential for the health and well-being of individuals. In this theory, the underlying psychological mechanism for the development of burnout is the feeling of lack of reciprocity in social exchange relations [11,14]. According to Farber [1,8-13], the way an individual copes with these feelings of frustration can lead to the development of one type of burnout or another. Consequently, subjects with "frenetic" burnout work increasingly harder to the point of exhaustion in search of success that is equal to the level of stress caused by their efforts. Workers with "underchallenged" burnout are presented with insufficient motivation and, given their talents and/or skills, have to cope with monotonous and unstimulating conditions that fail to provide the necessary satisfaction. Workers with "worn-out" burnout are those who give up when faced with stress or lack of gratification. This proposal for the classification of the syndrome was conceptualised and systematised from documentary analysis of Faber's clinical work [15] and its validity was explored [16] until a consistent and operative definition was reached [17]. The classification criterion for this typology is based on the level of dedication at work: high in "frenetic" subjects (active coping style), intermediate in "underchallenged" workers and low in "worn-out" subjects (passive coping style) [13,15,17].

"Frenetic" type burnout refers to a category of subjects who are very involved and ambitious and who overload themselves to fulfil the demands of their jobs. **"**Involvement" is the investment of all of necessary efforts until difficulties are overcome; "ambition" is the great need to obtain major successes and achievements and "overload" involves risking one's health and neglecting personal lives in the pursuit of good results [15-17]. This burnout profile is a category of exhausted but effective workers (at least in the short term), who are close to excessive commitment or even close to becoming workaholics. These people seem to develop the syndrome because they use up their energy resources on disproportionate dedication [15-21].

The "underchallenged" type of burnout refers to indifferent and bored subjects who fail to experience personal development in their jobs. "Indifference" is a lack of concern, interest and enthusiasm in work-related tasks; "boredom" describes one's experience of work as a monotonous, mechanical and routine experience with little variation in activities and "lack of development" is the desire by individuals to take on other jobs where they can better develop their skills [15-17]. "Underchallenged" subjects are exhausted but are more typified by their cynicism, owing to their loss of interest and the dissatisfaction they feel for tasks with which they do not identify, all of which are related with burnout [15-17,20,22-26].

The "worn-out" type refers to subjects who present with feelings of a lack of control over the results of their work and a lack of acknowledgement for their efforts, which finally leads them to neglect their responsibilities. "Lack of control" is the feeling of defencelessness or impotence as result of dealing with situations beyond their control; "lack of acknowledgement" is the belief that the organisations those individuals work for fail to take their efforts and dedication into account and "neglect" refers to the individual's disregard as a common response to most difficulties [15-17]. The "worn-out" profile, characterised by sluggish behaviour, is strongly associated with all of the dimensions of the definition by Maslach, Schaufeli and Leiter [2]. It is, therefore, the profile of exhausted, cynical and rather ineffective workers [15-17,20,27-30].

The work by Montero-Marín and García-Campayo shows how structural aspects, such as temporary work contracts, allow differences to be established between the described burnout types [17]. Temporary workers are seen to have a more frenetic attitude in general, while permanent employees are seen to have fewer challenges and more wear. To date, the possible associations between the different burnout types and other sociodemographic and occupational variables have not been studied. The purpose of this study is to examine the different general sociodemographic and occupational characteristics associated with burnout syndrome in other studies (such as age, gender, being in a stable relationship, having children, level of education, number of hours worked per week, occupation, length of service in an organisation, monthly income, contract duration and contract type) as elements that may be related to the different subtypes of burnout syndrome, in an attempt to identify the variables with the greatest predictive value for each profile.

The following points were considered specifically as working hypotheses: that a large number of hours worked per week, a factor traditionally associated with the development of burnout probably owing to the exhaustion it triggers [31-34], could have a particularly relevant weight in the "frenetic" subtype, given the significant degree of involvement, ambition and overload that characterises it; that those occupations involving monotonous and repetitive tasks traditionally associated with burnout as an antecedent factor [22,23,25] could be specifically related with the development of the "underchallenged" subtype given the indifference, boredom and lack of personal development experienced; and that the time worked in an organisation, a factor related with the development of the syndrome perhaps owing to the prolonged exposure to a system of contingencies that do not encourage satisfaction or commitment [35-37], could be more characteristic of the "worn-out" burnout subtype given the absence of control and acknowledgement, and the neglect felt by individuals in this situation. Shedding light on associations of this type would permit a better characterisation of these profiles and would facilitate the understanding and specific identification of subjects with burnout.

## Methods

### Study design

The correlation method was used with a cross-sectional design for data collection. However, attention was given to the development of variables over time so that any associations could be considered from a causal perspective [38]. The measurements were obtained by a self-reported online questionnaire completed by participants who had previously given their informed consent.

### Participants

The study population consisted of all employees of the University of Zaragoza working in January 2008 (N = 5,493). The required sample size was calculated so as to be able to make estimates with a 95% confidence level and a 3.5% margin for error, presuming an 18% prevalence of burnout [39], resulting in 427 subjects. The response rate expected in web-based surveys, based on past studies, was roughly 27% [40,41]. Therefore, 1,600 subjects were selected by means of random stratified sampling with proportional allocation depending on occupation (58% teaching and research staff or "TRS", 33% administration and service personnel or "ASP", 9% trainees or "TRA") from an alphabetical list of the entire workforce. The final sample consisted of n = 409 participants. This size exceeded the criterion suggested by Freeman whereby the number of participants must be greater than 10 (k+1), with k being the number of co-variables [42]. The sample size was therefore psychometrically adequate for the study. Sample size calculation and random sampling were performed with Epidat 3.1. software.

### Procedure

An e-mail was sent to the selected subjects explaining the aims of the research. This message contained a link to an online questionnaire and two access passwords for subjects to complete the questionnaire during the month of February 2008. All participants received an anonymous report with a correction and explanation of their results. This project was approved by the Ethics Committee of Aragon.

### Measurements

#### Sociodemographic and Occupational Factors

Subjects were first asked to complete a series of specifically prepared questions related to general sociodemographic and occupational characteristics. The questionnaire collected information on the variables of age, gender, whether or not the subject was in a stable relationship, children ("children" vs. "no children"), level of education ("secondary or lower", "university", "doctorate"), number of hours worked per week, occupation ("TRS", "ASP", "TRA"), length of service in years, monthly income, contract duration ("permanent" vs. "temporary") and contract type ("full-time" vs. "part-time").

#### Burnout Types

Subjects were then asked to complete the "Burnout Clinical Subtype Questionnaire" or BCSQ-36 (English version in Additional file 1 and Spanish version in Additional file 2) [17]. This questionnaire consists of 36 items distributed into 3 scales and 9 subscales. The "frenetic" scale consisted of the "involvement" (e.g., "I react to difficulties in my work with greater participation"), "ambition" (e.g., "I have a strong need for important achievements in my work") and "overload" (e.g., "I overlook my own needs to fulfil work demands") dimensions. The "underchallenged" scale consisted of the "indifference" (e.g., "I feel indifferent about my work and have little desire to succeed"), "lack of development" (e.g., "My work doesn't offer me opportunities to develop my abilities") and "boredom" (e.g., "I feel bored at work") dimensions. Finally, the "worn-out" scale consisted of the "neglect" (e.g., "When things at work don't turn out as well as they should, I stop trying"), "lack of acknowledgement" (e.g., "I think my dedication to my work is not acknowledged") and "lack of control" (e.g., "I feel the results of my work are beyond my control") dimensions. Subjects had to indicate the degree of agreement with each of the statements presented according to a Likert-type scale with 7 response options, scored from 1 (totally agree) to 7 (totally disagree). The scores for the scales were calculated as the sum of the scores obtained in their subscales. Results are presented in scalar scores. The internal consistency was: "frenetic" α = 0.84 ("involvement" α = 0.80, "ambition" α = 0.89, "overload" α = 0.86); "underchallenged" α = 0.92 ("indifference" α = 0.88, "lack of development" α = 0.88, "boredom" α = 0.86); "worn-out" α = 0.87 ("neglect" α = 0.86, "lack of acknowledgement" α = 0.88, "lack of control" α = 0.81). The convergence between the BCSQ-36 and MBI-GS questionnaires is adequate, given that the former provides a broader definition that is especially useful from a clinical perspective [17].

### Data analysis

The continuous sociodemographic and occupational variables were categorised into groups that were coherent with the original profile characterisations [1,8-13,15]. The former variables were introduced into the analysis as dummy variables as follows: age (<35, 35-50, >50), number of hours worked per week (<35 hours, 35-40 hours, >40 hours), length of service in years (<4 years, 4-16 years, >16 years), monthly income (under €1,200, €1,200-2,000, over €2,000. A general and by-occupation descriptive analysis was initially made of the participating subjects' sociodemographic and occupational features, using percentages to summarise the categorical variables and the χ^2 ^contrast test to assess differences in percentages. Means, standard deviations, medians, interquartile ranges and minimum and maximum values were utilised to describe the distribution of data collected using the BCSQ-36 scales and subscales.

Maslach and Jackson [43], followed by Maslach, Jackson and Leiter [44], considered burnout dimensions to be continuous variables. These variables could be used to express the degree of syndrome severity in three levels, namely low, intermediate and high, as a result of dividing the sample into three groups of equal size (33% of subjects), with each dimension classified according to the terciles. Among other criteria [45,46], a number of authors have interpreted these scores from a dichotomous point of view for the purpose of distinguishing those subjects with serious burnout symptoms from other individuals. Accordingly, it was suggested that the high scoring subjects would be those above the third quartile (25% of subjects) for each of the dimensions [47,48]. This approach was used in this study. The advantage of using this type of dichotomous criterion is that it also allows potential problems arising from small samples to be attenuated for subjects in the considered cases. Therefore, in the absence of previously established cut-off points for the BCSQ-36 with a clinical criterial benchmark, those participants situated above sample percentile 75 (P_75_) in each of the profiles (questionnaire scale scores) were defined as "high score" participants, whereas those situated below this level were considered "low score" participants in the variable "status" [47]. In the bivariate analysis, the possible association between the presence or absence of burnout types with each of the variables of interest was evaluated by means of a simple logistic regression (LR) model, which provided a raw odds ratio (OR), and its 95% confidence interval (CI) estimation. The statistical significance of the association was assessed using the Wald test.

Factors that gave a statistically significant result in the bivariate analysis (p < 0.05) were then included in a multivariate LR model. Estimates were provided for ORs adjusted for the variables included in the multivariate model and their 95% CIs. The statistical significance of adjusted ORs was evaluated using the Wald test. Linear trend p values were also calculated in those variables that had originally been measured continuously and had given significant results in the multivariate model. They were introduced into the model without being stratified. The fit of each multivariate model was evaluated with the Hosmer-Lemeshow χ^2 ^goodness-of-fit test, and its discriminatory power by means of the area below the ROC curve, taking into account the forecast probabilities and the variable status (high score/low score), with a cut-off point at *p *= 0.5. All of the tests were bilateral and were performed with a significance level of *p *< 0.05. Data analysis was performed with the SPSS-15 statistical software package.

## Results

### Characteristics of the study participants

The final sample consisted of n = 409 participants, which represents a response rate of 25.6%. The response rate was distributed as follows: 19.3% teaching and research staff, 36.5% administration and service personnel and 25.8% trainees. The mean age of participants was 40.51 years (SD = 9.09); 44.4% were males, and 21.9% were not in a stable relationship. A total of 42.9% worked as TRS, 46.9% as ASP and 10.2% were TRA. Table 1 shows the participants' general and by-occupation characteristics. The TRS group included subjects with higher qualifications and higher income (p < 0.001). The ASP group had the lowest number of work hours per week (p < 0.001). The TRA group was clearly different from the ASP and TRS groups, having the lowest age, the highest proportion of subjects with no children, the shortest length of service, no permanent contracts (p < 0.001) and the lowest prevalence of full-time work (p = 0.006).

**Table 1:** Sociodemographic and occupational characteristics of the participants

	TOTAL (n = 409)	TRS (n = 176)	ASP (n = 191)	TRA (n = 42)	*p*
**AGE**					< 0.001

**<35 years**	29.5%	23.8%	19.8%	97.6%	

**35-50 years**	57.0%	59.3%	66.8%	2.4%	

**>50 years**	13.5%	16.9%	13.4%	- -	

**SEX**					0.728

**male**	44.4%	42.4%	45.2%	48.8%	

**STABLE RELATIONSHIP**					0.456

**no**	21.9%	19.2%	23.4%	26.8%	

**CHILDREN**					<0.001

**no children**	50.1%	47.6%	42.3%	97.4%	

**EDUCATION**					<0.001

**secondary**	15.5%	0.6%	31.9%	2.5%	

**university**	52.1%	28.5%	65.4%	90.2%	

**doctorate**	32.4%	70.9%	2.7%	7.3%	

**N° OF WORKING HOURS**					<0.001

**<35 h/wk**	40.6%	16.8%	65.9%	22.5%	

**35-40 h/wk**	26.8%	24.8%	27.9%	30.0%	

**>40 h/wk**	32.6%	58.4%	6.2%	47.5%	

**LENGHT OF SERVICE**					<0.001

**<4 years**	18.5%	10.5%	12.2%	80.5%	

**4-16 years**	44.6%	49.4%	45.7%	19.5%	

**>16 years**	36.9%	40.1%	42.1%	- -	

**MONTHLY INCOME**					<0.001

**<€1,200**	31.1%	19.5%	26.1%	97.6%	

€**1,200-2,000**	42.1%	27.6%	66.3%	2.4%	

**>€2,000**	26.8%	52.9%	7.6%	- -	

**CONTRACT DURATION**					<0.001

**permanent**	63.6%	69.2%	72.3%	- -	

**CONTRACT TYPE**					0.006

**full-time**	93.8%	93.6%	96.3%	82.9%	

TRS = Teaching or Research Staff; ASp = Administration or Service Personnel; TRA = Trainees.

* *p *value for χ^2 ^contrast test

### Descriptive results

Table 2 shows the descriptive statistics for the BCSQ-36 scales and subscales. The highest scores were found for the "frenetic" subtype (Md = 4.12; SD = 0.80), followed by the "worn-out" subtype (Md = 3.79; SD = 0.90) and finally the "underchallenged" subtype (Md = 3.12; SD = 1.15), while dispersion values occurred in the reverse order from highest to lowest. The values from the scales did not occupy the entire range of possible responses, with special mention given to the minimum values for the involvement subscale (min = 2.00) and the maximum values for the neglect subscale (max = 5.50).

**Table 2:** Descriptive statistics for the BCSQ-36 scales and subscales (n = 409)

BCSQ-36	Md	SD	Mdn	**Q**_**1**_	**Q**_**3**_	min	max
***Frenetic sub-type***	4.12	0.80	4.00	3.58	4.58	2.25	7.00

**Involvement**	4.92	0.84	5.00	4.50	5.25	2.00	7.00

**Ambition**	3.91	1.20	3.75	3.00	4.75	1.00	7.00

**Overload**	3.53	1.29	3.25	2.75	4.50	1.00	7.00

***Underchallenged sub-type***	3.12	1.15	3.00	2.33	3.83	1.00	6.75

**Indifference**	2.58	1.20	2.50	1.75	3.00	1.00	7.00

**Boredom**	3.04	1.40	3.00	2.00	3.87	1.00	7.00

**Lack of Development**	3.73	1.37	3.50	3.00	4.56	1.00	7.00

***Worn-out sub-type***	3.79	0.90	3.83	3.17	4.33	1.33	6.42

**Lack of Control**	4.44	1.17	4.50	3.50	5.25	1.20	7.00

**Lack of Acknowledgement**	4.42	1.42	4.50	3.25	5.50	1.00	7.00

**Neglect**	2.52	0.90	2.75	2.00	3.00	1.00	5.50

Md = mean; SD = standard deviation; Mdn = median; Q_1_/Q_3 _= inter-quartile range; min/max = minimum and maximum score.

### Burnout type, sociodemographic and occupational risk factors

Table 3 shows the raw and adjusted ORs for the "frenetic" burnout type. Only the number of hours worked per week and the type of working hours showed statistical significance in the multivariate model for this profile. Specifically, those participants working more than 40 hours per week had a greater likelihood of having a high score than those who worked less than 35 hours per week (adjusted OR = 5.69; 95% CI = 2.52-12.82). In addition, those who worked part-time were more likely to have a high score than those in full-time employment (adjusted OR = 3.30; 95% CI = 1.12-9.74). The linear trend test for the number of hours worked per week provided a significant result (χ^2 ^= 22.56; p < 0.001). No significant differences were found between the observed and expected differences when the Hosmer-Lemeshow test was applied (χ^2 ^= 3.54; p = 0.896). The area under the ROC curve was 0.74 (95% CI = 0.68-0.80; p < 0.001).

**Table 3:** Sociodemographic and occupational risk factors for the "frenetic" type

FACTOR	high score (%)	low score (%)	raw OR (95% CI)	*p*	adjusted OR (95% CI)	*p*
**AGE**						

**>50 years**	9 (17.3)	43 (82.7)	ref.		ref.	

**35-50 years**	46 (20.2)	182 (79.8)	1.21 (0.55-2.65)	0.639	1.66 (0.65-4.26)	0.288

**<35 years**	48 (41.4)	68 (58.6)	3.37 (1.50-7.56)	**0.003**	2.94 (0.93-9.35)	0.067

**SEX**						

**female**	52 (23.5)	169 (76.5)	ref.		-	

**male**	51 (29.0)	125 (71.0)	1.33 (0.84-2.08)	0.219	-	-

**STABLE RELATIONSHIP**						

**yes**	78 (25.1)	233 (74.9)	ref.		-	

**no**	25 (29.1)	61 (70.9)	1.22 (0.72-2.08)	0.455	-	-

**CHILDREN**						

**1 or more**	37 (19.6)	152 (80.4)	ref.		ref.	

**none**	61 (32.3)	128 (67.7)	1.96 (1.22-3.14)	**0.005**	1.25 (0.68-2.32)	0.467

**EDUCATION**						

**secondary**	11 (18.3)	49 (81.7)	ref.		-	

**university**	60 (28.8)	148 (71.2)	1.81 (0.88-3.71)	0.107	-	-

**doctorate**	32 (24.8)	97 (75.2)	1.47 (0.68-3.16)	0.325	-	-

**HOURS PER WEEK**						

**<35 hours**	23 (15.1)	129 (84.9)	ref.		ref.	

**35-40 hours**	21 (20.8)	80 (79.2)	1.47 (0.77-2.83)	0.246	1.42 (0.65-3.10)	0.382

**>40 hours**	55 (44.7)	68 (55.3)	4.54 (2.57-8.01)	**<0.001**	5.69 (2.52-12.82)	**<0.001**

**OCCUPATION**						

**TRS**	52 (30.4)	119 (69.6)	ref.		ref.	

**ASP**	33 (17.8)	152 (82.2)	0.50 (0.30-0.82)	**0.006**	1.76 (0.81-3.81)	0.154

**TRA**	18 (43.9)	23 (56.1)	1.79 (0.89-3.60)	0.102	0.93 (0.34-2.55)	0.888

**LENGHT OF SERVICE**						

**<4 years**	29 (39.7)	44 (60.3)	ref.		ref.	

**4-16 years**	50 (28.2)	127 (71.8)	0.60 (0.34-1.06)	0.077	0.92 (0.40-2.09)	0.835

**>16 years**	24 (16.3)	123 (83.7)	0.30 (0.16-0.56)	**<0.001**	0.69 (0.22-2.13)	0.516

**MONTHLY INCOME**						

**>€2,000**	25 (24.0)	79 (76.0)	ref.		ref.	

**€1,200-2,000**	32 (19.3)	134 (80.7)	0.75 (0.42-1.36)	0.352	0.60 (0.26-1.42)	0.250

**<€1,200**	44 (36.4)	77 (63.6)	1.81 (1.01-3.23)	**0.047**	0.92 (0.32-2.65)	0.880

**CONTRACT DURATION**						

**Permanent**	47 (18.7)	205 (81.3)	ref.		ref.	

**Temporary**	56 (38.6)	89 (61.4)	2.74 (1.73-4.35)	**<0.001**	1.10 (0.49-2.49)	0.819

**CONTRACT TYPE**						

**full-time**	91 (24.5)	281 (75.5)	ref.		ref.	

**part-time**	12 (48.0)	13 (52.0)	2.85 (1.26-6.47)	**0.012**	3.30 (1.12-9.74)	**0.031**

% refer to the percentage in each stratum. Raw OR: Odds Ratio resulting from bivariate analysis. Adjusted OR: Odds Ratio for significant variables (p ≤ 0.05) in bivariate analysis through a multivariate logistic regression model. CI: confidence interval. Ref. = reference category. 'High score' implies scores higher than the upper quartile of the scores observed in the sample', 'low score' implies scores lower or equal than the upper quartile.

Table 4 shows the raw and adjusted ORs for the "underchallenged" burnout type. Only gender and occupation variables kept their statistical significance in the multivariate analysis for this profile. Specifically, the ASP group had a greater likelihood of having a high score than did the TRS group (adjusted OR = 2.85; 95% CI = 1.16-7.01), as did males compared to females (adjusted OR = 2.16; 95% CI = 1.31-3.55). No significant differences were found between the observed and expected differences for the multivariate model of the "underchallenged" profile when the Hosmer-Lemeshow test was applied (χ^2 ^= 2.83; p = 0.945). The area under the ROC curve was 0.68 (95% CI = 0.61-0.74; p < 0.001).

**Table 4:** Sociodemographic and occupational risk factors for the "underchallenged" type

FACTOR	high score (%)	low score (%)	raw OR (95% CI)	*p*	adjusted OR (95% CI)	*p*
**AGE**						

**>50 years**	12 (23.1)	40 (76.9)	ref.		-	

**35-50 years**	65 (28.5)	163 (71.5)	1.33 (0.66-2.69)	0.430	-	-

**<35 years**	26 (22.4)	90 (77.6)	0.96 (0.44-2.10)	0.924	-	-

**SEX**						

**female**	46 (20.8)	175 (79.2)	ref.		ref.	

**male**	57 (32.4)	119 (67.6)	1.82 (1.16-2.87)	**0.009**	2.16 (1.31-3.55)	**0.002**

**STABLE RELATIIONSHIP**						

**yes**	77 (24.8)	234 (75.2)	ref.		-	

**no**	26 (30.2)	60 (69.8)	1.32 (0.78-2.23)	0.306	-	-

**CHILDREN**						

**1 or more**	52 (27.5)	137 (72.5)	ref.		-	

**none**	48 (25.4)	141 (74.6)	0.90 (0.57-1.42)	0.641	-	-

**EDUCATION**						

**secondary**	22 (36.7)	38 (63.3)	ref.		ref.	

**university**	58 (27.9)	150 (72.1)	0.67 (0.36-1.22)	0.192	1.14 (0.57-2.27)	0.704

**doctorate**	23 (17.8)	106 (82.2)	0.37 (0.19-0.75)	**0.005**	1.74 (0.56-5.41)	0.340

**HOURS PER WEEK**						

**<35 hours**	49 (32.2)	103 (67.8)	ref.		ref.	

**35-40 hours**	28 (27.7)	73 (72.3)	0.81 (0.46-1.40)	0.445	0.89 (0.49-1.61)	0.695

**>40 hours**	20 (16.3)	103 (83.7)	0.41 (0.23-0.73)	**0.003**	0.61 (0.29-1.27)	0.187

**OCCUPATION**						

**TRS**	27 (15.8)	144 (84.2)	ref.		ref.	

**ASP**	65 (35.1)	120 (64.9)	2.889 (1.73-4.81)	**<0.001**	2.85 (1.16-7.01)	**0.023**

**TRA**	11 (26.8)	30 (73.2)	1.956 (0.87-4.37)	0.102	2.64 (0.89-7.83)	0.079

**LENGHT OF SERVICE**						

**<4 years**	15 (20.5)	58 (79.5)	ref.		-	

**4-16 years**	44 (24.9)	133 (75.1)	1.28 (0.66-2.48)	0.466	-	-

**>16 years**	44 (29.9)	103 (70.1)	1.65 (0.85-3.22)	0.141	-	-

**MONTHLY INCOME**						

**>2000€**	21 (20.2)	83 (79.8)	ref.		ref.	

**1200-2000€**	52 (31.3)	114 (68.7)	1.80 (1.01-3.22)	**0.047**	1.29 (0.60-2.79)	0.512

**<1200€**	30 (24.8)	91 (75.2)	1.30 (0.69-2.45)	0.412	1.01 (0.41-2.50)	0.987

**CONTRACT DURATION**						

**Permanent**	72 (28.6)	180 (71.4)	ref.		-	

**Temporary**	31 (21.4)	114 (78.6)	0.68 (0.42-1.10)	0.117	-	-

**CONTRACT TYPE**						

**full-time**	99 (26.6)	273 (73.4)	ref.		-	

**part-time**	4 (16.0)	21 (84.0)	0.52 (0.18-1.57)	0,249	-	-

% refer to the percentage in each stratum. Raw OR: Odds Ratio resulting from bivariate analysis. Adjusted OR: Odds Ratio for significant variables (p ≤ 0.05) in bivariate analysis through a multivariate logistic regression model. CI: confidence interval. Ref. = reference category. 'High score' implies scores higher than the upper quartile of the scores observed in the sample', 'low score' implies scores lower or equal than the upper

Table 5 shows the raw and adjusted ORs for the "worn-out" burnout type. Statistical significance was found in the multivariate model for the length of service in the organisation, being in a stable relationship, children and level of education. Subjects who had been working between four and sixteen years were more likely to have a high score (adjusted OR = 3.44; 95% CI = 1.34-8.86), as were those with more than sixteen years of service (adjusted OR = 4.56; 95% CI = 1.47-14.16), when compared to those who had worked for fewer than four years. This result was also the case with workers who were not in stable relationships compared to those who were (adjusted OR = 1.91; 95% CI = 1.05-3.45) and in those who did not have children compared to those who did (adjusted OR = 1.90; 95% CI = 1.09-3.31). However, those subjects with a university education showed a lower likelihood of this type of burnout compared to those with only secondary education or lower (adjusted OR = 0.48; 95% CI = 0.24-0.96). The linear trend test for the length of service showed a significant result (χ^2 ^= 4.84; p = 0.028). No significant differences were found between the observed and expected differences when the Hosmer-Lemeshow test was applied (χ^2 ^= 8.37; p = 0.301). The area under the ROC curve was 0.70 (95% CI = 0.64-0.76; p < 0.001).

**Table 5:** Sociodemographic and occupational risk factors for the "worn-out" type

FACTOR	high score (%)	low score (%)	raw OR (95% CI)	*p*	adjusted OR (95% CI)	*p*
**AGE**						

**>50 years**	21 (40.4)	31 (59.6)	ref.		ref.	

**35-50 years**	72 (31.6)	156 (68.4)	0.68 (0.37-1.27)	0.225	0.87 (0.44-1.76)	0.707

**<35 years**	24 (20.7)	92 (79.3)	0.38 (0.19-0.79)	**0.009**	0.80 (0.30-2.13)	0.654

**SEX**						

**female**	68 (30.8)	153 (69.2)	ref.		-	

**male**	50 (28.4)	126 (71.6)	0.89 (0.58-1.38)	0.609	-	-

**STABLE RELATIONSHIP**						

**yes**	79 (25.4)	232 (74.6)	ref.		ref.	

**no**	39 (45.3)	47 (54.7)	2.44 (1.48-4.00)	**<0.001**	1.91 (1.05-3.45)	**0.033**

**CHILDREN**						

**1 or more**	47 (24.9)	142 (75.1)	ref.		ref.	

**none**	65 (34.4)	124 (65.6)	1.58 (1.01-2.47)	**0.043**	1.90 (1.09-3.30)	**0.024**

**EDUCATION**						

**secondary**	27 (45.0)	33 (55.0)	ref.		ref.	

**university**	48 (23.1)	160 (76.9)	0.37 (0.20-0.67)	**0.001**	0.48 (0.24-0.95)	**0.037**

**doctorate**	43 (33.3)	86 (66.7)	0.61 (0.33-1.14)	0.123	0.60 (0.30-1.19)	0.146

**HOURS PER WEEK**						

**<35 hours**	41 (27.0)	111 (73.0)	ref.		-	

**35-40 hours**	28 (27.7)	73 (72.3)	1.04 (0.59-1.82)	0.896	-	-

**>40 hours**	38 (30.9)	85 (69.1)	1.21 (0.72-2.04)	0.475	-	-

**OCCUPATION**						

**TRS**	54 (31.6)	117 (68.4)	ref.		-	

**ASP**	57 (30.8)	128 (69.2)	0.96 (0.62-1.51)	0.876	-	-

**TRA**	7 (17.1)	34 (82.9)	0.45 (0.19-1.07)	0.071	-	-

**LENGHT OF SERVICE**						

**<4 years**	8 (11.0)	65 (89.0)	ref.		ref.	

**4-16 years**	55 (31.1)	122 (68.9)	3.66 (1.64-8.15)	**0.001**	3.44 (1.34-8.86)	**0.010**

**>16 years**	55 (37.4)	92 (62.6)	4.86 (2.17-10.88)	**<0.001**	4.56 (1.47-14.16)	**0.009**

**MONTHLY INCOME**						

**>2000€**	34 (32.7)	70 (67.3)	ref.		-	

**1200-2000€**	56 (33.7)	110 (66.3)	1.05 (0.62-1.76)	0.860	-	-

**<1200€**	26 (21.5)	95 (78.5)	0.56 (0.31-1.02)	0.060	-	-

**CONTRACT DURATION**						

**Permanent**	86 (34.1)	166 (65.9)	ref.		ref.	

**Temporary**	32 (22.1)	113 (77.9)	0.55 (0.34-0.87)	**0.012**	1.04 (0.52-2.05)	0.919

**CONTRACT TYPE**						

**full-time**	113 (30.4)	259 (69.6)	ref.		-	

**part-time**	5 (20.0)	20 (80.0)	0.57 (0.21-1.56)	0.277	-	-

% refer to the percentage in each stratum. Raw OR: Odds Ratio resulting from bivariate analysis. Adjusted OR: Odds Ratio for significant variables (p ≤ 0.05) in bivariate analysis through a multivariate logistic regression model. CI: confidence interval. Ref. = reference category. 'High score' implies scores higher than the upper quartile of the scores observed in the sample', 'low score' implies scores lower or equal than the upper quartile.

## Discussion

This study is the first to our knowledge that suggests the existence of associations between the different burnout subtypes (classified according to the degree of dedication to work) and the different sociodemographic and occupational characteristics that are congruent with the definition of each of the subtypes. The results of this work assist the clinical differentiation of subtypes by introducing sociodemographic and occupational variables into the differential burnout model as specific risk factors that are easy to identify. They also facilitate an understanding of the clinical phenomenology of the profiles, encouraging future working hypotheses of a causal nature to be considered among the variables and enabling more specific interventions to be developed for the syndrome.

The variables "number of hours worked per week" and "contract type" showed significance in the adjusted model for the "frenetic" burnout subtype. Those employees who invested more than forty hours per week in their jobs had a greater risk of presenting this type of burnout compared to those working fewer than thirty five hours. The number of hours worked per week was associated directly and linearly with the "frenetic" burnout sub-type in such a way that when the number of hours was increased, so was the risk of developing this burnout profile. This variable seems to be the key factor in the configuration of this profile and could contribute to the development of the syndrome by increasing worker exhaustion levels [15,17,31-34]. Data regarding contract type show that workers in part-time employment present a higher risk of having this burnout subtype compared to full-time employees. This result may seem contradictory, but this is not the case when we consider that these subjects tend to have several jobs at the same time (e.g., adjunct lecturers and students on traineeships), which is associated with burnout syndrome in general [49]. All of these results are consistent with what has been put forward in the qualitative works to which we previously referred [1,8-12,15] and they enable the rapid identification of the burnout profile of concern to us. The significance of guilt feelings in the development and continuation of burnout syndrome [6,7] has already been pointed out. Specifically, the "frenetic" subtype feels guilt when faced with the prospect of not achieving set goals, given the ambition and great need for achievement that characterise subjects with this profile [1,15]. These individuals adopt active coping strategies and invest all of their possible efforts until they become exhausted and overloaded [17]. Consequently, the treatment for this profile requires a holistic approach that takes into account the cause of their excessive ambition and their associated guilty feelings, in addition to a reduction of their involvement and lessening of their dedication to work in the interest of satisfying their personal needs.

On the other hand, the variables "occupation" and "gender" were statistically significant in the adjusted model for the "underchallenged" burnout subtype. In our study, the ASP group had a greater likelihood of developing this burnout profile when compared to the TRS group. Burnout can generally occur in all types of occupational groups [50], but public assistance jobs, such as those performed by ASP group members, seem to have an increased risk [51]. This risk is possibly due to the antecedent effect produced by the characteristics of this type of work [22,23,25,26]. It is necessary to take the degree of worker satisfaction into consideration with respect to the characteristics of their jobs in order to address their discontent [52], as dissatisfied workers present a greater risk of suffering from burnout [31,32,53]. 33]. It is also important to pay attention to worker preferences with regard to the type of work they would like to perform [54], given that a sustained organisational policy concerning these values improves satisfaction levels and reduces absenteeism in the long term [55]. With regard to "gender", our study has found that males are at greater risk of suffering from "underchallenged" burnout than females, perhaps owing to the fact that the role of males has always been linked to social expectations of professional development [47]. Generally, employees with the "underchallenged" profile have to cope with the disenchantment caused by feeling trapped in an occupational activity to which they are indifferent, which bores them and produces no gratification. These employees present a cynical attitude [17] and are invaded by guilty feelings due to the ambivalence they feel for their work and by their desire for change. These people have lost their objectivity with respect to their natural right to experience needs for personal development and to try to pursue them [9,15]. Basic components of treatments for this clinical profile should include restoring balance to this distorted view of their needs by approaching the associated guilty feelings, encouraging a renewal of interest and personal development at work by presenting job-related tasks in a significant light.

Lastly, "length of service", "level of education", "stable relationships" and "having children" were significant factors in the adjusted model for the "worn-out" burnout subtype. Employees with between four and sixteen years of service in the organisation and those with more than sixteen year of service were at greater risk of developing the "worn-out" profile in comparison with those with fewer than four years of service. "Length of service" in the organisation showed a direct linear association with the "worn-out" type, to the extent that the longer the service, the greater the likelihood of having this burnout profile. This variable has a certain ambivalence in its relationship with burnout syndrome in general, given that associations have been found that are both direct [35], inverse [31] and even absent [56]. This contradiction may be due to the differential impacts of the various types of organisations on their employees [57,58] and to the personal relations and forms of communication established in the workplace [36], some of which offer protection from the development of the syndrome, while others induce it. Having a university degree, together with a stable relationship and the presence of children, was seen to be factors that protect from the "worn-out" burnout subtype, which is in line with results obtained in other studies for burnout syndrome in general [33,34,50,53]. Our results suggest that the prolonged exposure to the environment provided by the organisation that was the object of our study turned out to be a significant risk factor for developing the helplessness characterising the "worn-out" profile. Employees with this profile adopt a passive coping strategy, becoming ineffective in performing work tasks and they feel guilty because they do not fulfil the responsibilities of their post [10,15,17]. For this subtype, consideration is given to the suitability of treating not only the feelings of despair, passive coping and inefficacy that characterise it, but also of intervening in the actual contingency system of the organisation, directing its influence as much as possible towards developing commitment to tasks and encouraging the establishment of a social support network.

Through the analysis of the ROC curves, we have seen that the performance shown by the considered sociodemographic and occupational factors in predicting burnout types is superior to a random classifier. Nevertheless, they are far from being the ideal classifier, which means that it might be worth considering other variables that may be associated with the burnout subtypes, such as personality features or specific coping strategies. We should also not overlook the fact that as values for the considered variables were self-reported, they may have been influenced by socially-desirable responses. This phenomenon may have occurred more particularly in the subscales of involvement and neglect, as dedication to work is quite important in Western culture, dedication to work. Further, given that the minimum values for the former and the maximum values for the latter do not encompass the entire range of possible responses. On the other hand, the cross-sectional design of the study forces us to be cautious when drawing conclusions regarding the aetiology of burnout subtypes. However, confirmation of these types of associations does not come under the scope of this study. The main aim of this work was to ascertain in an exploratory fashion which sociodemographic and occupational variables could be associated with the different burnout subtypes in order to assist in the recognition and understanding of these clinical profiles. This goal does not require that the established associations must be of a causal nature. Nevertheless, the fact that these sociodemographic and occupational variables existed prior to the time of measurement (which implies the fulfilment of the premise of temporal precedence) and evidence of a dose-response relationship (statistically significant p values for linear trend analysis) would support that hypothesis. Therefore, our study makes advancement possible in the generation of new hypotheses that may be subsequently confirmed by means of a suitable research design [38]. With regard to the representative nature of the sample, we believe that although the response rate obtained may seem low and the distribution by occupational levels may seem uneven, these values are comparable to those found in other studies using the same data collection procedures [40,41]. We consider that one strength of this study lies in the fact that the work was carried out with a broad and multi-occupational sample of university employees in positions with very different characteristics, which reinforces the possibility of generalising our conclusions. Additionally, data quality was controlled by eliminating possible errors in the questionnaire transcription process through the use of purpose-designed software.

## Conclusions

Our results add to the understanding of the type of professional burnout present in employees of a university organisation in Spain and support the idea of a differential characterisation of burnout syndrome by providing specific associations with a number of sociodemographic and occupational factors that are congruent with the definition by clinical profiles. We have seen that the "frenetic" profile is highly associated with the number of hours per week dedicated to work, that the "underchallenged" profile is related with the type of occupation and that the "worn-out" profile is associated with the cumulative effect over time of the characteristics of an organisation. The recognition of these variables will assist the process of clinical differentiation of those affected by the syndrome, as these are factors that can be rapidly identified. These subtypes of burnout will need to be taken into account when designing specific treatments according to the characteristics of each subject if we are to increase the effectiveness of our interventions for burnout syndrome.

## Competing interests

The authors declare that they have no competing interests.

## Authors' contributions

JMM, JGC, MG and FM conceived the project. JMM and JMC collected the data. JMM, MFP and SG conducted the statistical analysis, and all authors interpreted the results, drafted the manuscript and read and approved the final manuscript.

## Pre-publication history

The pre-publication history for this paper can be accessed here:

http://www.biomedcentral.com/1471-244X/11/49/prepub

## Supplementary Material

Additional file 1 **English version of the "Burnout Clinical Subtype Questionnaire" (BCSQ-36)**. Click here for file

Additional file 2 Spanish version of the "Burnout Clinical Subtype Questionnaire" (BCSQ-36) Click here for file
