# Supplementary material for: Sociodemographic and occupational risk factors associated with the development of different burnout types: the cross-sectional University of Zaragoza study
Source: BMC Psychiatry. 2011 Mar 29;11:49. doi: 10.1186/1471-244X-11-49 (PMC3074532; doi:10.1186/1471-244X-11-49)
Supplement: Additional file 1 — English version of the "Burnout Clinical Subtype Questionnaire" (BCSQ-36). [file 1471-244X-11-49-S1.DOC]

The following is a series of statements indicating experiences that may occur at work. Read each statement carefully and mark with an X the option that best represents how you feel, what you do and what you think about your work. There are no right or wrong answers. Please **DO NOT LEAVE ANY STATEMENT UNANSWERED.**

|  | **Totally disagree** | **Strongly disagree** | **Disagree** | **Unsure** | **Agree** | **Strongly agree** | **Totally**  **agree** |
| --- | --- | --- | --- | --- | --- | --- | --- |
| 1. I need to achieve great success in my work | O | O | O | O | O | O | O |
| 2. I think the dedication I invest in my work is more than what I should for my health | O | O | O | O | O | O | O |
| 3. I invest the necessary effort in my work until I overcome difficulties | O | O | O | O | O | O | O |
| 4. I am ambitious to obtain important results in my work | O | O | O | O | O | O | O |
| 5. I neglect my personal life when I pursue important achievements in my work | O | O | O | O | O | O | O |
| 6. I get very involved in solving work-related problems | O | O | O | O | O | O | O |
| 7. I feel the need to achieve important goals in my work | O | O | O | O | O | O | O |
| 8. I risk my health when I pursue good results in my work | O | O | O | O | O | O | O |
| 9. If I don’t achieve the expected result in my work, I try harder to achieve it | O | O | O | O | O | O | O |
| 10. I have a strong need for important achievements in my work | O | O | O | O | O | O | O |
| 11. I overlook my own needs to fulfil work demands | O | O | O | O | O | O | O |
| 12. I react to difficulties in my work with greater participation | O | O | O | O | O | O | O |
| 13. I feel indifferent about my work and have little desire to succeed | O | O | O | O | O | O | O |
| 14. I would like to be doing another job that is more challenging for my abilities | O | O | O | O | O | O | O |
| 15. I feel my work is mechanical and routine | O | O | O | O | O | O | O |
| 16. I have little interest for the tasks involved in my job | O | O | O | O | O | O | O |
| 17. I feel that my work is an obstacle to the development of my abilities | O | O | O | O | O | O | O |
| 18. My work offers me little variety in its activities | O | O | O | O | O | O | O |
| 19. I’m not enthusiastic about my work | O | O | O | O | O | O | O |
| 20. I would like to be doing another job where I can better develop my talents | O | O | O | O | O | O | O |
| 21. I am unhappy with my work because the tasks involved are monotonous | O | O | O | O | O | O | O |
| 22. I behave in a unconcerned and reluctant way at work | O | O | O | O | O | O | O |
| 23. My work doesn’t offer me opportunities to develop my abilities | O | O | O | O | O | O | O |
| 24. I feel bored at work | O | O | O | O | O | O | O |
| 25. The people who need my services don’t show appreciation or gratitude for my efforts | O | O | O | O | O | O | O |
| 26. When things at work don’t turn out as well as they should, I stop trying | O | O | O | O | O | O | O |
| 27. I feel helpless in many situations in my work | O | O | O | O | O | O | O |
| 28. Professional recognition doesn’t depend on efforts made at work | O | O | O | O | O | O | O |
| 29. I give up in response to difficulties in my work | O | O | O | O | O | O | O |
| 30. I feel defenseless in some situations in my work | O | O | O | O | O | O | O |
| 31. The organisation I work for doesn’t take notice of effort and dedication | O | O | O | O | O | O | O |
| 32. I give up in the face of any difficulties in my work tasks | O | O | O | O | O | O | O |
| 33. I feel the results of my work are beyond my control | O | O | O | O | O | O | O |
| 34. I think my dedication to my work is not acknowledged | O | O | O | O | O | O | O |
| 35. When the effort I invest in work is not enough, I give in | O | O | O | O | O | O | O |
| 36. I deal with many situations in my work that are beyond my control | O | O | O | O | O | O | O |

**Scoring**

“Frenetic” subscale is made up of three dimensions: ‘involvement’ (items nº 3, 6, 9, 12), ‘ambition’ (items nº 1, 4, 7, 10) and ‘overload’ (items nº 2, 5, 8, 11); “underchallenged” subscale includes the following three dimensions: ‘indifference’ (items nº 13, 16, 19, 22), ‘lack of development’ (items nº 14, 17, 20, 23) and ‘boredom’ (items nº 15, 18, 21, 24); and wornout subscale is made up of three dimensions: ‘neglect’ (items nº 26, 29, 32, 35), ‘lack of acknowledgement (items nº 25, 28, 31, 34) and ‘lack of control’ (items nº 27, 30, 33, 36). Answers are scored from 1 (completely agree) to 7 (completely disagree).
