# Supplementary material for: Sociodemographic and occupational risk factors associated with the development of different burnout types: the cross-sectional University of Zaragoza study
Source: BMC Psychiatry. 2011 Mar 29;11:49. doi: 10.1186/1471-244X-11-49 (PMC3074532; doi:10.1186/1471-244X-11-49)
Supplement: Additional file 2 — Spanish version of the "Burnout Clinical Subtype Questionnaire" (BCSQ-36) [file 1471-244X-11-49-S2.DOC]

A continuación se presentan una serie de enunciados que indican vivencias que puede experimentar en el trabajo. Lea cada frase con atención y señale con una X la opción que mejor represente cómo se siente, lo que hace o lo que piensa respecto a su actividad laboral. No existen respuestas correctas o incorrectas. Por favor, **NO DEJE NINGUNA RESPUESTA SIN CONTESTAR**.

|  | **Totalmente en desacuerdo** | **Muy en desacuerdo** | **En desacuerdo** | **Indeciso** | **De acuerdo** | **Muy de acuerdo** | **Totalmente de**  **acuerdo** |
| --- | --- | --- | --- | --- | --- | --- | --- |
| 1. Tengo la necesidad de obtener grandes triunfos en el trabajo | O | O | O | O | O | O | O |
| 2. Creo que invierto más de lo saludable en mi dedicación al trabajo | O | O | O | O | O | O | O |
| 3. En el trabajo, invierto todo el esfuerzo necesario hasta superar las dificultades | O | O | O | O | O | O | O |
| 4. Ambiciono la obtención de grandes resultados en el trabajo | O | O | O | O | O | O | O |
| 5. Descuido mi vida personal al perseguir grandes objetivos en el trabajo | O | O | O | O | O | O | O |
| 6. Me involucro con gran esfuerzo en la solución de los problemas del trabajo | O | O | O | O | O | O | O |
| 7. Siento la necesidad de abordar grandes metas en el trabajo | O | O | O | O | O | O | O |
| 8. Arriesgo mi salud en la persecución de buenos resultados en el trabajo | O | O | O | O | O | O | O |
| 9. Si en el trabajo no logro el resultado esperado, me empeño más para alcanzarlo | O | O | O | O | O | O | O |
| 10. Tengo una fuerte necesidad de grandes logros en el trabajo | O | O | O | O | O | O | O |
| 11. Ignoro mis propias necesidades por cumplir con las demandas del trabajo | O | O | O | O | O | O | O |
| 12. Ante las dificultades en el trabajo reacciono con mayor participación | O | O | O | O | O | O | O |
| 13. Me siento indiferente y con poca inclinación hacia mi trabajo | O | O | O | O | O | O | O |
| 14. Me gustaría dedicarme a otro trabajo que planteara mayores desafíos a mi capacidad | O | O | O | O | O | O | O |
| 15. Siento que mi trabajo es mecánico y rutinario | O | O | O | O | O | O | O |
| 16. Tengo poco interés por las tareas de mi puesto de trabajo | O | O | O | O | O | O | O |
| 17. Siento que mi actividad laboral es un freno para el desarrollo de mis capacidades | O | O | O | O | O | O | O |
| 18. Mi trabajo me ofrece poca variedad de actividades | O | O | O | O | O | O | O |
| 19. No tengo ilusión por mi actividad laboral | O | O | O | O | O | O | O |
| 20. Me gustaría desempeñar otro trabajo en el que pudiera desarrollar mejor mi talento | O | O | O | O | O | O | O |
| 21. Estoy descontento en mi trabajo por la monotonía de las tareas | O | O | O | O | O | O | O |
| 22. En el trabajo me comporto con despreocupación y desgana | O | O | O | O | O | O | O |
| 23. Mi trabajo no me ofrece oportunidades para el desarrollo de mis aptitudes | O | O | O | O | O | O | O |
| 24. Me siento aburrido en el trabajo | O | O | O | O | O | O | O |
| 25. Quienes demandan mi servicio no muestran aprecio ni gratitud por mis esfuerzos | O | O | O | O | O | O | O |
| 26. Cuando las cosas del trabajo no salen del todo bien dejo de esforzarme | O | O | O | O | O | O | O |
| 27. Me siento impotente en muchas situaciones de mi trabajo | O | O | O | O | O | O | O |
| 28. El reconocimiento profesional no depende de lo que uno se esfuerce en el trabajo | O | O | O | O | O | O | O |
| 29. Me rindo como respuesta a las dificultades en el trabajo | O | O | O | O | O | O | O |
| 30. Me siento indefenso ante algunas situaciones de mi trabajo | O | O | O | O | O | O | O |
| 31. En la organización donde trabajo no se tienen en cuenta el esfuerzo y la dedicación | O | O | O | O | O | O | O |
| 32. Abandono ante cualquier dificultad en las tareas de mi trabajo | O | O | O | O | O | O | O |
| 33. Siento que se escapan de mi control los resultados de mi trabajo | O | O | O | O | O | O | O |
| 34. Pienso que mi dedicación en el trabajo no se ve reconocida | O | O | O | O | O | O | O |
| 35. Cuando el esfuerzo invertido en el trabajo no es suficiente, me doy por vencido | O | O | O | O | O | O | O |
| 36. En mi trabajo trato con muchas situaciones que están fuera de mi control | O | O | O | O | O | O | O |
